# Malocclusion and Scoliosis: Is There a Correlation?

**DOI:** 10.3390/jpm13081249

**Published:** 2023-08-10

**Authors:** Sabina Saccomanno, Stefano Saran, Licia Coceani Paskay, Nicola Giannotta, Rodolfo Francesco Mastrapasqua, Alessio Pirino, Fabio Scoppa

**Affiliations:** 1 Orthodontic Residency, Department of Life, Health and Environmental Sciences, University of L’Aquila, 67100 L’Aquila, Italy; sabinasaccomanno@hotmail.it; 2 Department of Human Sciences, Innovation and Territory, School of Dentistry, University of Insubria, 21100 Varese, Italy; ngiannotta@studenti.uninsubria.it; 3 Academy of Orofacial Myofunctional Therapy (AOMT), 910 Via De La Paz, Ste.106, Pacific Palisades, CA 90272, USA; lcpaskay@gmail.com; 4 ENT Department, Rivoli Hospital, ASL TO3, 10098 Rivoli, Italy; rfmastrapasqua@aslto3.piemonte.it; 5 Department of Biomedical Sciences, University of Sassari, 07100 Sassari, Italy; axelpir@uniss.it; 6 Chinesis I.F.O.P. Osteopathy School, Faculty of Medicine and Dental Surgery, University of Rome “Sapienza”, 00185 Rome, Italy; fabioscoppa@chinesis.org

**Keywords:** scoliosis, malocclusion, survey, TMJ, orofacial pain, tongue posture

## Abstract

Introduction: Scoliosis is a complex three-dimensional malformation of the spine. Although its etiology is still being investigated, it is clear that a number of factors can influence this syndrome. The spinal deformity of idiopathic scoliosis can be viewed from an etiopathogenetic perspective as a symptom of a complicated condition with a multifactorial etiology. Numerous studies have established its relationship with malocclusion, but it is still unclear how these factors interact. Malocclusion is a change in the physiological alignment of the upper and lower teeth that can be either dental or skeletal in origin. This study’s objective is to assess the relationship between scoliosis and malocclusion. Material and Methods: A total of 646 patients were enrolled (554 females and 92 males), 447 with scoliosis and 199 without, from private dental and orthopedic practices, to answer an anonymous questionnaire. They were selected in private dental and orthopedic practices where they had dental and orthopedic examinations. Twenty-two patients were excluded because of a lack of answers. Participants were given a bilingual survey, in English and Italian, composed of 13 questions formulated specifically for this study, using Google Forms (Google LLC, Mountain View, CA, USA). Results: Univariate analysis of the question “Do you have scoliosis?” shows a significant correlation with the following questions: “Was scoliosis a family issue?” (*p* < 0.05 OR 7.30 IC: 3.05–17.46) “Do you have malocclusion?” (*p* < 0.05, OR: 1.19 IC:1.0–1.34) and “Was mal-occlusion a family issue?” (*p* < 0.01, OR: 1.39 IC 1.10–1.77). Performing a multivariate analysis for the same variables, the best predictors of scoliosis were “Was scoliosis a family issue?” (*p* < 0.001) and “Was malocclusion a family issue?” (*p* < 0.05), while the question “Do you have malocclusion” lost significance. Conclusion: This study adds further confirmation that there might be an important connection between malocclusion and scoliosis; it suggests that dentists and orthopedists have to check, as early as possible, for the probable presence of both pathologies to avoid a severe progression which, in most cases, may require significant therapy and even surgery.

## 1. Introduction

Idiopathic scoliosis is a deformity of the spine that primarily affects previously healthy children, predominantly girls, during a growth spurt (Weinstein et al.) [1]. The evidence that idiopathic scoliosis is a complex three-dimensional deformity of the spine, rather than a simple lateral curvature, has been well described in the following studies: Roaf [2]; Pedriolle and Vidal [3]; Pedriolle, Becchetti, Vidal and Lopez [4]; Deacon, Flood and Dickson [5]; Dickson [6]; Stagnara [7]; and Pedriolle [4]. Those suffering from scoliotic deformities with typical vertebral rotation in the thoracic and lumbar spine showed a significant decrease in thoracic kyphosis and an increase in lumbar lordosis (Inoue et al.) [8]. Lateral views show that the displaced segments of the spine are always an extension, even when kypho-scoliosis is present (Perdriolle et al.) [9].

Recent studies also focus on the significance of the scoliosis component in the sagittal plane and its role in the pathogenetic evolution of idiopathic scoliosis (Schlosser et al.) [10]. Furthermore, morphological changes in the scoliotic vertebrae appear to be related to the sagittal spinal profile in adolescents with idiopathic scoliosis (Pasha et al.) [11].

The main diagnostic criterion is a coronal curvature exceeding 10 degrees on an anterior–posterior X-ray. The severity of scoliosis is expressed by the Cobb angle. This condition has been divided into three types: infantile (presenting from birth to 3 years), juvenile (presenting from 3 to 10 years) and adolescent (presenting from 10 years to skeletal maturity (Pedriolle et al.) [12].

However, structural scoliosis can be seen with a Cobb angle under 10° (Xiong et al.) [13] with a potential for progression. Progression is more common in girls during the growth spurt at puberty, referred to as progressive idiopathic scoliosis (Negrini et al.) [14].

By definition, idiopathic scoliosis is of unknown etiology: clinical history, clinical analysis and radiological examinations do not provide clear evidence for any specific origin (Machida et al.) [15]. From an etiopathogenetic point of view, therefore, the spinal deformity caused by idiopathic scoliosis may be defined as a sign of a syndrome with a multifactorial etiology (Negrini et al.) [14], confirming what was already described by Brooks et al. in 1975 [16].

If progressive scoliosis remains untreated, it can create several problems, even life-threatening ones, by developing pulmonary conditions, chronic pain and drastic changes in quality of life [14].

The link between scoliosis and dental malocclusion is still controversial. There are several articles that have studied this association (Laskowska et al.) [17]; (Lippold et al.), [18], (Saccucci et al.) [19], but there is not enough evidence about the correlation and the etiology (Perez Belloso et al.) [20]. Malocclusion is an abnormal relationship between the teeth on the upper and the lower arches (dental) and, in some situations, between the jaws (skeletal). A malocclusion frequently appears during the growth period, especially in Western societies: therefore, a dental patient can simultaneously be an orthopedic patient as well. The sagittal malocclusion is usually defined by Angle’s class one (I), class two (II) with divisions 1 and 2, and class three (III). From a skeletal point of view, Angle’s class II is characterized by a mandible that is posteriorly located (retrusion) or poorly developed. A dental class II can be divided into two types: division 1 and division 2. Division 1 is when the upper incisors are tilted outwards, creating significant overjet, or when there is a significant sagittal distance between the upper and the lower incisors. Division 2 is characterized by what is called a “deep bite” or an excessive vertical overlap of the maxillary central incisors over the mandibular central incisors. A dental class III is characterized by the mandible and lower teeth being in an advanced position compared to the maxillary teeth and can be characterized by a cross-bite that can be anterior or lateral [21]. In other words, the palatal dental arch always needs to be “larger” than the mandibular dental arch. This proportion is completely inverted in a class III, and when this proportional relationship is partial or monolateral or bilateral but not frontal, it gives raise to cross-bites. A monolateral cross-bite determines, in almost all cases, a dysfunctional movement of the jaw, as the teeth are not properly allied to slide past each other and to protect each other, resulting in the stifled growth and movement of the crossed teeth.

Some articles have investigated which malocclusion is more likely linked to scoliosis. It appears that asymmetrical malocclusions could favor or be favored by scoliosis, although the direction of the influence (ascending or descending) is still not clear. It has been reported [22] that a cross-bite, and in particular the monolateral type, is the type of malocclusion most connected to scoliosis. An orthodontist should be able to diagnose malocclusion and correct it, especially while the patient is in the growing phase [23]. Furthermore, according to some authors, it seems that transversal malocclusions are the malocclusions more related to scoliosis and the worsening of it. Moreover, a few authors suggest that a deep bite and other vertical abnormalities appear in patients with various spinal pathologies; not just scoliosis, but also with those who have a pelvic tilt and pelvic torsion. Other studies report that a deviated midline and asymmetries of the mandible are tied to the severity of the scoliosis, but it is not clear which is the main etiological factor or even if there is a common one that cause both illnesses [24]. Some studies have reported that a class II malocclusion occurs in scoliosis patients more often than in patients with a healthy spine curvature [24,25].

This study analyzes if there is a link between temporo-mandibular disorders, scoliosis and malocclusion. Furthermore, the questionnaire asked if the patients had relatives with scoliosis to understand if there is a possible genetic predisposition. The patients were asked if they had any previous orthodontic treatment to see if there is any biological or timing connection between pathologies and orthodontics. Moreover, it was asked if patients had been informed about the possible and probable connection between occlusion, spinal posture and growth. The purpose of this article is to analyze a significant number of patients with and without scoliosis and malocclusion to identify any possible associations between scoliosis and malocclusion, as reported by those patients who filled out the questionnaire.

## 2. Material and Methods

A total of 646 patients were enrolled (554 females and 92 males), 447 with scoliosis and 199 without. They were selected in private dental and orthopedic practices where they had dental and orthopedic examinations. Twenty-two patients were excluded because of a lack of answers. Patients were given a bilingual survey, in English and Italian, composed of 13 questions formulated specifically for this study, using Google Forms (Google LLC, Mountain View, CA, USA) and accessible online (Table 1). The questionnaire was shared online through various emails. All collected data were anonymized and identified only by an ID and a time stamp. There were no reminders transmitted to patients to help them feel free to answer. It was specified that the purpose of the questionnaire was to find ways for clinicians to improve their skills in treating patients. It was ensured that each patient provided one answer by controlling the timing and different kinds of responses. The patients were asked to complete the questionnaire without any possible compensation or benefit in return. The questionnaire was compiled specifically for this study, and due to the contingency of the COVID-19 pandemic waves, pre-testing was not a possible option. All participants signed informed consent and accepted the privacy policy for the protection of their personal data before completing the survey. No personal information that could identify the individuals was collected and the data were analyzed in aggregate form only. All data points are expressed as absolute frequency (percentage). Dichotomic correlations of data were assessed using the Fisher exact test while age groups were compared by using the Mann–Whitney U test and logistic regression. Considering the worldwide prevalence of malocclusion of 56%, we anticipated a minimum difference of 14% in prevalence, an alpha error of 0.05 and beta of 0.2; thus, we calculated the sample size for dichotomic variables and established 186 responders per group [26].

## 3. Results

A total of 646 patients responded to the survey, but 22 of them were excluded for missing more than four responses. The questions and results of the survey are compiled in Table 2.

In Table 3, univariate analysis of the question, “Do you have scoliosis?” shows a significant correlation with the following questions: “Was scoliosis a family issue?” (*p* < 0.05 OR 7.30 IC: 3.05–17.46), “Do you have malocclusion?” (*p* < 0.05, OR: 1.19 IC:1.0–1.34) and “Was malocclusion a family issue?” (*p* < 0.01, OR: 1.39 IC 1.10–1.77). Performing a multivariate analysis for the same variables, the best predictors of scoliosis were “Was scoliosis a family issue?” (*p* < 0.001) and “Was malocclusion a family issue?” (*p* < 0.05), while the question “Do you have malocclusion” lost significance.

A univariate analysis was performed considering the questions: “Did your orthopedist inform you about the possibility that scoliosis/posture and malocclusion can influence each other?” and “Did your dentist inform you about the possibility that malocclusion could influence the spine and your posture?”, correlating them with current age, age at diagnosis and the following questions: “Was malocclusion a family issue?”, “Did you suffer from TMJ (mandibular) pain?”, “Did you suffer from TMJ pain?” and “ Did your scoliosis appear before, after or while the orthodontic therapy?”, but we found no correlations.

For the question “What kind of malocclusion do you have”, the answer “Deviated mandible” showed a higher prevalence in patients who reported having scoliosis (*p* < 0.05, OR: 2.67 IC 1.01–7.67) compared to those who did not. However, during the analysis of the answers, this question was deemed to have been too confusing in its formulation and was dropped from the final results.

## 4. Discussion

Scoliosis is still a disabling disease today and if not diagnosed early it can lead to serious complications. Early screening for scoliosis is desirable and prevents patients from longer and more complex treatments and spinal surgery. There are still only a few peer-reviewed studies linking scoliosis to malocclusion. Huggare et al. [22] and Lippold et al. [18] reported the relationship between idiopathic scoliosis and facial asymmetry or malocclusions with a transverse discrepancy such as cross-bites. A study by Saccucci et al. [19] reported a higher incidence of malocclusions in individuals with scoliosis compared with the group of healthy subjects. According to Laskowska M. et al., [17], the incidence of malocclusions is greater in children with idiopathic scoliosis than in healthy ones. This result is in accordance with the results of this study. However, according to Langella F. et al. [27], there is evidence from low-quality studies suggesting an increased prevalence of occlusal dysfunction in patients with known spinal deformity, but the conclusions have a high risk of bias. No evidence of beneficial effects of orthodontic treatment on spinal deformity was found. Lippold et al. [18] reported a predisposition to cross-bites in scoliotic individuals. It was interesting to note that the three most reported types of malocclusion in our study were the generic crowded teeth (31%), followed by deep bite (21%) and then overjet (14%), confirming some data in the literature, but cross-bites were mentioned only by 9% of the responders. Unfortunately, currently, this connection is not known by most orthodontists, orthopedists and doctors, who treat these two kinds of pathologies, although the clinicians treating postural problems, myofascial therapists, cranio-osteopathic physicians and those who have some training in these issues have been clinically aware of this link for decades.

Does this lack of awareness have an explanation? The mouth and the spine seem to be two distant systems but, in a clinical setting, they are more intertwined than one might think. For example, a recent study [28] focused on the role played by the temporomandibular joint and dental occlusion on the balance of the mother’s body and on the muscular forces reflected during childbirth labor.

It is possible to believe that the study of the relationship between occlusion and spine, and between occlusion and body posture, should be encouraged because the topic has great clinical relevance among various health professionals, as it could improve the quality of care for patients with scoliosis and/or malocclusion [28].

The role of the tongue and swallowing also plays a crucial role in the etiopathogenesis of malocclusion. The tongue, along with correct swallowing, shapes the palate and dental arches and, at the neurological level, during swallowing, the tongue activates the widely distributed receptors of the cortical and subcortical areas [24,25,28]. A lower habitual posture of the tongue and the consequent narrow palate can lead to respiratory problems impacting physiological nasal breathing. To compensate for these respiratory problems, the patient changes their head and neck position. The tongue posture is also very important because it is connected to the hyoid bone that is itself connected to various cervical muscles. This can, according to different studies, determine a change in body posture [23], and therefore it is important for the orthodontist to correct the tongue position to help patients’ optimize breathing and posture. Tongue position can even be altered by a restricted frenulum [24]. In these cases, the first approach may be either orofacial myofunctional therapy or surgery (tongue-tie release) or a combination of the two (surgery preceded and followed by orofacial myofunctional therapy). These minor treatments can be of substantial benefit to scoliotic patients.

One of the first steps to helping reduce or slow the progression of scoliosis and malocclusion is to educate patients and professionals about this specific correlation to reach an early and correct diagnosis. According to the answers of this questionnaire, people are not aware of the connection between scoliosis and malocclusion, but neither do the orthodontists or the specialists in charge of managing the scoliosis themselves, as 42% of responders mentioned having scoliosis before the orthodontic treatment, 83% responded that the orthopedist did not mention connections between scoliosis and malocclusion and a similar percentage (86%) mentioned that the orthodontist did not mention any connection between orthodontic treatment and scoliosis. Therefore, it is very important to educate patients with scoliosis about the need to consult with an orthodontist, and educate patients with significant malocclusion about the need to see a spine specialist, because it is probable that the same patient would present both issues. An early diagnosis is very important in both problems because it would help avoid invasive surgical therapies often used to treat scoliosis or severe skeletal malocclusions. It appears from the results or our questionnaire that not only is there a link between malocclusion and scoliosis, but those patients with scoliosis have a higher possibility of having temporomandibular/orofacial pain disorders as well, as 43% of the responders indicated they did have TMJD/orofacial pain. Moreover, it is useful for patients with TMJD/orofacial pain problems to receive an assessment of their posture and spinal condition along with their dental occlusion. Conversely, it would be very helpful for medical doctors who treat TMJ/orofacial pain disorders to assess spinal posture, which can contribute to and increase the severity of symptoms, because of all the muscular connections between the cervical spine and the temporomandibular area.

For this reason, occlusions like the unilateral cross-bite or the asymmetrical class II should be promptly treated in patients with scoliosis to avoid worsening of the spinal curvature. The results of this questionnaire suggest that both conditions are present in other family members, even considering the limited awareness the patients might have had about the health history of their family members. This awareness of family history can be helpful for both diagnosis and prevention because, if scoliosis or malocclusions are present in several members of the family, a patient should be advised to be more proactive and to avoid or minimize the onset of one or both conditions. Since scoliosis is not always evident and is asymptomatic in the beginning, it is prudent to check the spinal posture and alignment before the beginning of any orthodontic treatment, as sometimes parents and relatives mistakenly think that orthodontic therapy can cause scoliosis.

The current study investigated if patients with scoliosis also presented malocclusion in significant numbers, but future studies will be needed to establish a more specific relationship between the two disorders, or even a causal relationship, as a cause–effect relationship is not currently defined. In order to establish this relationship, tighter collaboration between orthodontists and orthopedists is needed to gather relevant data.

It makes sense for orthodontists to spear-head this collaboration in search of the specific relationships between malocclusion and scoliosis, because they are the ones who are more likely to see young children. An orthodontist works with children to take advantage of the growth and development spurts. If an orthodontist has easy protocols and tools available to assess scoliosis, then its damaging effects may be prevented or limited.

Conversely, if the orthopedist is aware of the connections between scoliosis and malocclusion, then the two professionals may be able to work in tandem, as both professionals are aware of part of the situation. Cross-education and using common assessment tools would allow them to better serve the young patient, who could be diagnosed and treated in tandem, as opposed to sequentially. Additional professionals may be involved as needed, such a PT or a posturologist, working during the orthodontic treatment.

One possible explanation on why this collaboration between orthodontists and orthopedists is still not happening is that scoliosis may be difficult to detect/diagnose in its early stages and postural instruments and tools may be expensive and not widely available, so there is still a need for an inexpensive solution. Currently, diagnosis for scoliosis (the Cobb angle) requires X-rays, which are controversial and therefore are not advised as a first approach.

Ideally, at the very least, there is a need for a common/reciprocal way to assess scoliosis and malocclusion. A proposal for an easy-to-use, multidisciplinary protocol for assessment of both malocclusion and scoliosis could be the Adam Forward Bending Test (Adobor et al., 2011) [29], a simple and inexpensive method, that, although not infallible, is the most used test in scoliosis research worldwide (Komang-Agung, Dwi-Purnomo, and Susilowati, 2017; Gashaw, Janakiraman and Belay, 2021) [30,31,32] and has been for decades (Wang, Ye & Wu, 1996) [32].

It is reasonable to conduct further studies about the possible importance of the Adam Forward Bending Test as a diagnostic tool for orthodontists. It could help those professionals in detecting the signs of scoliosis and making the proper referral, while familiarity with the Angle’s classification of malocclusion on the part of orthopedists could be helpful to the dual approach of these disorders in children and may establish if and which one leads to the other one.

Overall, it is right to mention some limitations of this study. By its very nature, a questionnaire involves personal perceptions, personal knowledge and opinion of a certain subject. And some people might have not been aware of the significance of some questions, or the intended meaning of some answers. Moreover, another limitation of the questionnaire was that among the types of malocclusion listed, the answer *Other* probably replaced the answer *Class III*, which was missing among the options. The question: *Is scoliosis a family issue?* might have been difficult to understand as well, especially if the respondents were young.

## 5. Conclusions

Considering all the findings of this study and the limitations of a questionnaire, it is still possible to reaffirm the correlation between scoliosis and oral malocclusion. This study suggests the necessity of assessing the spinal condition in patients with a diagnosed malocclusion, as well as checking for certain types of malocclusions in patients with a possible or confirmed presence of scoliosis. This study, which included a significant number of patients, offers an important contribution to the research about this pathological link between two systems that seem distant and disconnected but which are more intertwined than was previously assumed.

It seems clear that, since orthodontists and orthopedics are complementary and neither have all the answers, there is a current necessity to include malocclusion and scoliosis assessments in each other’s evaluation or assessment protocols, as the current collaboration between professionals is missing to the detriment of the patients’ health.

## Figures and Tables

**Table 1:** Demographic information.

Ranges of Age	Present Age
5–12	8
10–13	48
14–18	96
19–29	156
30–39	141
40–49	91
50–59	22
60+	22
Did not respond	10
**Ranges of Age**	**At what age did you notice scoliosis? (Only for patients with scoliosis)**
5–12	92
10–13	180
14–18	98
19–29	20
30–39	8
40–49	3
50–59	2
60+	1
Did not respond	27
**Ranges of Age**	**At what age did you have an orthodontic therapy?**
5–12	30
10–13	107
14–18	71
19–29	27
30–39	15
40–49	13
50–59	2
60+	2
Did not respond	75

**Table 2:** Survey answers.

Gender		
Female	554	90.3%
Male	67	8.6%
Did not respond	6	1.3%
**Malocclusion**		
Yes	466	72.2%
No	150	25.9%
Did not respond	11	2.4%
**Scoliosis**		
Yes	447	85.2%
No	177	14.3%
**Was scoliosis a family issue?**		
Yes	202	42.6%
No	253	53.4%
Unsure	19	4.0%
**Did your orthopedist inform you about the possibility that scoliosis/posture and malocclusion can influence each other?**		
Yes	60	12.7%
No	394	83.1%
Unsure	20	4.2%
**Did your dentist/orthodontist inform you about the possibility that malocclusion could influence the spine and your posture?**		
Yes	55	11.6%
No	409	86.3%
Unsure	10	4.2%
Did you have an orthodontic therapy before you noticed scoliosis? *		
Yes	163	63.6%
No	285	36.4%
* Limited to those who have scoliosis		
**Did you notice that, after scoliosis had appeared, your teeth became misaligned?**		
No	218	57.1%
Yes	124	32.5%
Unsure	15	3.9%
Did not respond	25	6.5%
**Did your scoliosis appear before, after or during the orthodontic therapy?**		
Before	202	42.6%
While	63	13.3%
After	72	15.2%
Did not respond	137	28.9%
**Did you suffer from TMJ (mandibular) pain?**		
No	255	53.8%
Yes	205	43.2%
Unsure	14	2.4%
**Do you feel the jaw is deviated in the same direction of the main scoliotic curve?**		
No	247	52.1%
Yes	114	24.1%
Unsure/Did not respond	113	23.8%
**What kind of malocclusion**		
Crowded teeth	169	31.3%
Deep bite	115	21.3%
Overjet	76	14.1%
Cross-bite	51	9.4%
Deviated mandible	30	4.8%
Little mandible (micrognathia)	21	3.3%
Other	71	11.3%
**What kind of orthodontic device did you use?**		
Fixed	161	34.0%
Mobile	82	17.3%
Both	72	15.2%
Aligners	21	4.4%
None	10	1.6%

* Limited to those who have scoliosis.

**Table 3:** Results.

	Significance	OR (IC)
Do you have scoliosis		
**Do you have malocclusion**	*p* < 0.05	1.19 (1.0–1.34)
**Was scoliosis a family issue**	*p* < 0.05	7.30 (3.05–17.46)
**Was malocclusion a family issue**	*p* < 0.01	1.39 (1.10–1.77)
**Did you suffer from TMJ pain**	N.S	0.96 (0.71–1.3)

## Data Availability

The corresponding author can share the data upon request.
